# Supplementary material for: Harnessing digital health to objectively assess cognitive impairment in people undergoing hemodialysis process: The Impact of cognitive impairment on mobility performance measured by wearables
Source: PLoS One. 2020 Apr 20;15(4):e0225358. doi: 10.1371/journal.pone.0225358 (PMC7170239; doi:10.1371/journal.pone.0225358)
Supplement: Attachment — Submitted filename: Response Letter_2020.3.26.docx [file pone.0225358.s001.docx]

Authors’ response to the academic editor

| Original Title | Harnessing Digital Health to Objectively Assess Cognitive Impairment in People undergoing Hemodialysis Process: The Impact of Cognitive Impairment on Mobility Performance Measured by Wearables |
| --- | --- |
| Manuscript ID | PONE-D-19-30554 |

3/26/2020

Dear Editor:

We appreciate the constructive comments and suggestions made by the academic editor. Where relevant, we have made changes that we believe are responsive to comments, and which enhance the quality of the manuscript.

Specific modifications to the manuscript in response to comments by the academic editor are highlighted using Word Text Highlight Color in the manuscript, and the updated locations in the manuscript are specified.

Overall, in the revised draft we shortened the Introduction section as requested by the reviewer #1. In response to the editor’s request, we publicly shared the database. We have also clarified that Hamad Medical Corporation or HMC is a non-profit educational organization. It is the principal public healthcare provider in the State of Qatar. None of the co-authors, including co-authors affiliated with HCM, claimed any relevant conflict of interest.

Thank you for your attention and consideration. We look forward to your final review.

Sincerely,

He Zhou, Fadwa Al-Ali, Changhong Wang, Abdullah Hamad, Rania Ibrahim, Tala Talal, Bijan Najafi

**Journal Requirements**

**C1.** When submitting your revision, we need you to address these additional requirements.

Please ensure that your manuscript meets PLOS ONE's style requirements, including those for file naming. The PLOS ONE style templates can be found at

http://www.journals.plos.org/plosone/s/file?id=wjVg/PLOSOne_formatting_sample_main_body.pdf and http://www.journals.plos.org/plosone/s/file?id=ba62/PLOSOne_formatting_sample_title_authors_affiliations.pdf.

**Response:** Done

**C2.** In your Data Availability statement, you have not specified where the minimal data set underlying the results described in your manuscript can be found. PLOS defines a study's minimal data set as the underlying data used to reach the conclusions drawn in the manuscript and any additional data required to replicate the reported study findings in their entirety. All PLOS journals require that the minimal data set be made fully available. For more information about our data policy, please see http://journals.plos.org/plosone/s/data-availability.

Upon re-submitting your revised manuscript, please upload your study’s minimal underlying data set as either Supporting Information files or to a stable, public repository and include the relevant URLs, DOIs, or accession numbers within your revised cover letter. For a list of acceptable repositories, please see http://journals.plos.org/plosone/s/data-availability#loc-recommended-repositories. Any potentially identifying patient information must be fully anonymized.

Important: If there are ethical or legal restrictions to sharing your data publicly, please explain these restrictions in detail. Please see our guidelines for more information on what we consider unacceptable restrictions to publicly sharing data: http://journals.plos.org/plosone/s/data-availability#loc-unacceptable-data-access-restrictions. Note that it is not acceptable for the authors to be the sole named individuals responsible for ensuring data access.

We will update your Data Availability statement to reflect the information you provide in your cover letter.

**Response:** The de-identified data was uploaded in a public repository: Data Archiving and Networking Services (DANS). <https://doi.org/10.17026/dans-xy5-n8c8>

Relevant DOI has been added into the revised cover letter.

**C3.** Please provide an amended statement that declares *all* the funding or sources of support (whether external or internal to your organization) received during this study, as detailed online in our guide for authors at http://journals.plos.org/plosone/s/submit-now. Please also include the statement “There was no additional external funding received for this study.” in your updated Funding Statement.

**Response:** Done

**Page 22 Line 435:** “*Support was provided by the Qatar National Research Foundation (Award number: NPRP 7-1595-3-405 and NPRP 10-0208-170400). There was no additional external funding received for this study. The content is solely the responsibility of the authors and does not necessarily represent the official views of the sponsor.*”

**C4.** Please include your amended Funding Statement within your cover letter. We will change the online submission form on your behalf.

**Response:** Done.

**C5.** We note that one or more of the authors are employed by a commercial company: 'Hamad Medical Corporation'.

Please provide an amended Funding Statement declaring this commercial affiliation, as well as a statement regarding the Role of Funders in your study. If the funding organization did not play a role in the study design, data collection and analysis, decision to publish, or preparation of the manuscript and only provided financial support in the form of authors' salaries and/or research materials, please review your statements relating to the author contributions, and ensure you have specifically and accurately indicated the role(s) that these authors had in your study. You can update author roles in the Author Contributions section of the online submission form.

Please also include the following statement within your amended Funding Statement.

“The funder provided support in the form of salaries for authors [insert relevant initials], but did not have any additional role in the study design, data collection and analysis, decision to publish, or preparation of the manuscript. The specific roles of these authors are articulated in the ‘author contributions’ section.”

If your commercial affiliation did play a role in your study, please state and explain this role within your updated Funding Statement.

**Response:** Please note that Hamad Medical Corporation (HMC) is a non-profit public healthcare provider in the State of Qatar (<https://www.hamad.qa/EN>). None of the co-authors, including authors affiliated with HMC, claimed any conflict of interest.

**C6.** Please also provide an updated Competing Interests Statement declaring this commercial affiliation along with any other relevant declarations relating to employment, consultancy, patents, products in development, or marketed products, etc.

Within your Competing Interests Statement, please confirm that this commercial affiliation does not alter your adherence to all PLOS ONE policies on sharing data and materials by including the following statement: "This does not alter our adherence to PLOS ONE policies on sharing data and materials.” (as detailed online in our guide for authors http://journals.plos.org/plosone/s/competing-interests) . If this adherence statement is not accurate and there are restrictions on sharing of data and/or materials, please state these. Please note that we cannot proceed with consideration of your article until this information has been declared.

* Please include both an updated Funding Statement and Competing Interests Statement in your cover letter. We will change the online submission form on your behalf.

**Response:** As indicated above, none of the co-authors claimed any conflict of interest. HMC is the principal public healthcare provider in the State of Qatar. It is a non-profit organization.

**Reviewer 1**

**R1C1.** I would only recommend authors to streamline introduction, that, as it is now, reads quite long.

**Response:** We refined the Introduction section in the revised manuscript to be more focusing.
